# Editorial: Behavior and Welfare of the Individual Within Large, Commercially-Relevant Groups

**DOI:** 10.3389/fvets.2021.656236

**Published:** 2021-03-23

**Authors:** Michael J. Toscano, Rebecca K. Meagher, Dana L. M. Campbell

**Affiliations:** ^1^Center for Proper Housing: Poultry and Rabbits (ZTHZ), Division of Animal Welfare VPH Institute, University of Bern, Bern, Switzerland; ^2^Dalhousie University, Halifax, NS, Canada; ^3^Agriculture and Food, Commonwealth Scientific and Industrial Research Organisation (CSIRO), Armidale, NSW, Australia

**Keywords:** welfare, tracking, precision livestock farming, livestock, agriculture, data analytics

Individuality is increasingly recognized as important and has become a focus of exploration in many fields that study behavior and other responses. Specifically in Animal Welfare where a variety of responses (e.g., behavioral, endocrinological, immunological, neurological, etc.) must be amalgamated comprehensively as animals adapt to stressors, animal individuality should be considered essential. The shift toward a focus on individuals (from group-level observations) has largely been driven by a growing awareness that differences between individuals can be consistent over time and across varied settings (1–4). With the increased attention toward individuality and its incorporation into our understanding of Animal Welfare, we should be encouraging and utilizing the most modern and advanced methods at our disposal. The current Research Topic comprises 10 articles that utilize novel methods to assess animal behavior, often using advanced sensor technologies and analytics, and answer questions about its relationship to welfare and physiological functioning.

Measuring the behavior of individuals in their current housing environments should consider long-lasting impacts of previous environments. Campbell et al. looked at the impacts of different types of rearing enrichments on subsequent ranging behavior in free-range hens. Radio-frequency identification technology was used to track individuals across a flock cycle to show that the type of environment they were reared in, affected how much they utilized the range area as adults with rearing perching structures increasing range usage.

The impacts of rearing environments as well as subsequent individual variation in ranging behavior on hen welfare was further explored by Bari et al.. Regular external measures of bird welfare such as body weight, plumage coverage, and comb wounds showed that hen welfare was affected by their rearing environment with control-reared hens showing worse feather coverage with age. There was also a relationship with range use where hens that spent more time outside had better feather coverage.

Developmental impacts on behavior can even precede the rearing environment, occurring via maternal stress and hormone deposition in chicken eggs. Peixoto et al. assessed offspring of stressed hens for anxiety-like and fear responses. The behavioral tests demonstrated limited impacts of maternal stressors and impacts did not appear to be caused by corticosterone depositions. The greatest differences were found among varying genotypes demonstrating the importance of considering the overarching influence of animal strains on individual variation in behavior when drawing general conclusions based on a specific breed.

While a focus on the individual is critical for direct understanding of behavior and welfare impacts, livestock animals are typically housed in groups and will thus be affected by those individuals surrounding them. Keshavarzi et al. analyzed GPS data to demonstrate that naïve cattle in paddocks with a virtual fence were facilitated by their herd mates when learning the correct responses to this new technology that signals the presence of an invisible fence line via audio and electrical cues.

Advances in sensor technology and computational abilities often require novel statistical methods to ensure generated data is used most effectively. In an example of how large complex datasets can be analyzed using these novel methods, McVey et al. examined the sequential order of dairy cows entering the milking parlor over a 6-month period using entropy as a tool to assess variance and the relationship to social and temporal correlates. Interestingly, cows at the front and rear of the queue proved to be most consistent, a feature reminiscent of classical concepts of dominance hierarchies. Further analysis using machine learning proved effective in relating the variables with productivity, health status, and behavior within the home pen.

Similarly, Chopra et al. examined the structural consistency of a commercial dairy herd using remote identification of pairs of cows within 3 meters of each other for more than 60 s. Proximity networks were then related to feeding-specific areas and the entire barn, as well as health status and productivity factors. The study employed applied network visualization and social network analysis to determine that associations of dyads were non-normal but rather related temporally and to specific barn areas.

Baur et al. described the development of a radiography protocol for assessing keel bone health in conscious laying hens. The radiographs distinguished between different types of fracture. They demonstrated that keel bone fractures are likely more common than was previously recognized, with 97% of the 150 birds studied having at least one fracture, showing that this method, which is easier to use on farm than other scanning technology, provides more detail and likely greater sensitivity than the common approach of physical palpation.

Bone health in poultry is an example of how health outcomes can be linked with individual developmental and psychosocial factors. Rokavec and Šemrov thus hypothesized that low body weight and high fear or stress would associate with bone condition. Although they did not find that low body weight or indicators of fearfulness and corticosterone levels in the feathers of younger birds predisposed them to bone deviations or fractures, such bone damage during lay was associated with lower concurrent open field activity and higher later sociality, posing questions about the causal relationships between putative indicators of fear, stress, social behavior and physical health in this species.

van der Zande et al. meanwhile, tested new behavioral analyses to quantify what they considered Dynamic Indicators of Resilience in pigs: measures of consistency in physical activity levels, automatically detected by ear tag accelerometers on piglets injected with Porcine Reproductive and Respiratory Syndrome Virus (PRRSV). They found that changes in skewness from pre-to post-injection predicted mortality risk, and that statistical tendencies suggested possible relationships between high variation in activity, as measured by the Root Mean Square Error (RMSE) in the days after the injection and its increase from pre-exposure levels, and clinical illness symptoms. This novel use of accelerometer data thus holds some promise for quantifying individuals' resilience to health challenges.

Finally, individual monitoring of behavior can provide insight into changes in the brain, as demonstrated by Armstrong et al. who tracked over 400 hens using RFID technology and correlated this with responses to standardized behavioral tests and brain samples from a subset of the hens. The results showed that increased ranging behavior may stimulate cell proliferation, a measure of plasticity, in the rostral hippocampus, thus contributing to the cognitive benefits of outdoor access, and that overall proliferation correlated with a personality indicator, tonic immobility as a fear response.

Overall, this body of research highlights the use of technology for precise data collection at the level of the individual and novel analysis methods to better understand factors that affect animal behavior and welfare as well as how these individuals are influenced by the groups they are housed within.

## Author Contributions

All authors listed have made a substantial, direct and intellectual contribution to the work, and approved it for publication.

## Conflict of Interest

The authors declare that the research was conducted in the absence of any commercial or financial relationships that could be construed as a potential conflict of interest.
